# Blockage of Spontaneous Ca^2+^ Oscillation Causes Cell Death in Intraerythrocitic *Plasmodium falciparum*

**DOI:** 10.1371/journal.pone.0039499

**Published:** 2012-07-06

**Authors:** Masahiro Enomoto, Shin-ichiro Kawazu, Satoru Kawai, Wakako Furuyama, Tohru Ikegami, Jun-ichi Watanabe, Katsuhiko Mikoshiba

**Affiliations:** 1 Laboratory for Developmental Neurobiology, RIKEN Brain Science Institute, Wako-shi, Saitama, Japan; 2 National Research Center for Protozoan Diseases, Obihiro University of Agriculture and Veterinary Medicine, Obihiro, Hokkaido, Japan; 3 Laboratory of Tropical Medicine and Parasitology, Dokkyo Medical University, Mibu, Tochigi, Japan; 4 Institute of Medical Science, University of Tokyo, Minato-ku, Tokyo, Japan; 5 Japan Science and Technology Agency, International Cooperative Research Project and Solution-Oriented Research for Science and Technology, Calcium Oscillation Project, Saitama, Japan; Université Pierre et Marie Curie, France

## Abstract

Malaria remains one of the world’s most important infectious diseases and is responsible for enormous mortality and morbidity. Resistance to antimalarial drugs is a challenging problem in malaria control. Clinical malaria is associated with the proliferation and development of *Plasmodium* parasites in human erythrocytes. Especially, the development into the mature forms (trophozoite and schizont) of *Plasmodium falciparum* (*P. falciparum*) causes severe malaria symptoms due to a distinctive property, sequestration which is not shared by any other human malaria. Ca^2+^ is well known to be a highly versatile intracellular messenger that regulates many different cellular processes. Cytosolic Ca^2+^ increases evoked by extracellular stimuli are often observed in the form of oscillating Ca^2+^ spikes (Ca^2+^ oscillation) in eukaryotic cells. However, in lower eukaryotic and plant cells the physiological roles and the molecular mechanisms of Ca^2+^ oscillation are poorly understood. Here, we showed the observation of the inositol 1,4,5-trisphospate (IP_3_)-dependent spontaneous Ca^2+^ oscillation in *P. falciparum* without any exogenous extracellular stimulation by using live cell fluorescence Ca^2+^ imaging. Intraerythrocytic *P. falciparum* exhibited stage-specific Ca^2+^ oscillations in ring form and trophozoite stages which were blocked by IP_3_ receptor inhibitor, 2-aminoethyl diphenylborinate (2-APB). Analyses of parasitaemia and parasite size and electron micrograph of 2-APB-treated *P. falciparum* revealed that 2-APB severely obstructed the intraerythrocytic maturation, resulting in cell death of the parasites. Furthermore, we confirmed the similar lethal effect of 2-APB on the chloroquine-resistant strain of *P. falciparum*. To our best knowledge, we for the first time showed the existence of the spontaneous Ca^2+^ oscillation in *Plasmodium* species and clearly demonstrated that IP_3_-dependent spontaneous Ca^2+^ oscillation in *P. falciparum* is critical for the development of the blood stage of the parasites. Our results provide a novel concept that IP_3_/Ca^2+^ signaling pathway in the intraerythrocytic malaria parasites is a promising target for antimalarial drug development.

## Introduction

**Figure 1:**
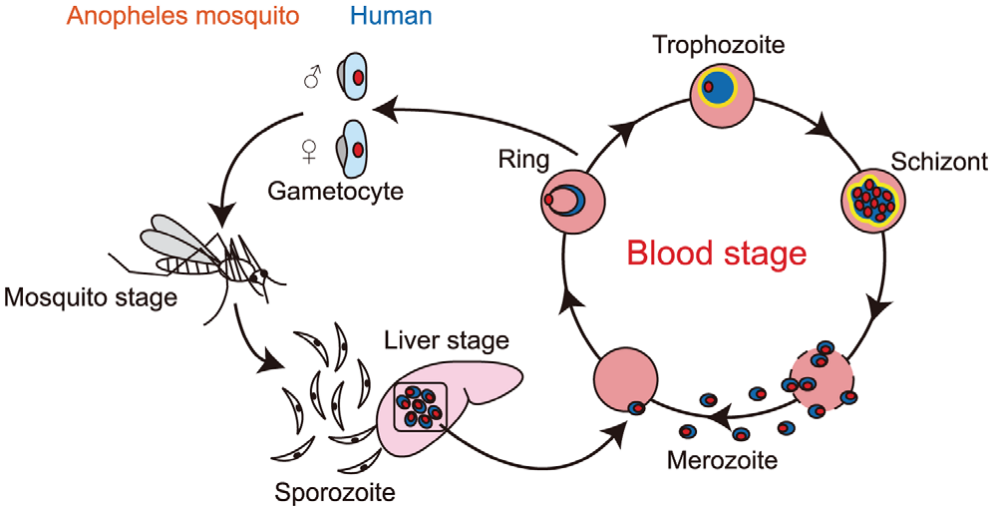
Life cycle of *Plasmodium. falciparum.* Schematic illustration of the life cycle of *P. falciparum*. The blood stages on which this study is focused are shown in detail.

Malaria continues to be a worldwide public health problem causing significant morbidity and mortality and its resistance to existing antimalarial drugs is a growing problem [1]. The life cycle of *Plasmodium* species is complex (Fig. 1). Infection of humans begins with a small inoculum of sporozoites from the salivary glands of a blood-feeding *Anopheles* mosquito. Sporozoites penetrate liver cells, transform and multiply asexually to produce thousands of free merozoites (liver stage). Each of these asexual merozoites invades an erythrocyte and enters into another phase of asexual reproduction, and then bursts the cell, releasing 8–32 more merozoites to invade more erythrocytes (blood stage). In infected erythrocytes, development of the parasites is accompanied by morphological changes such as ring form, trophozoite and schizont stages. *P falciparum* is responsible for the lethal form of human malaria. The mature forms of the intraerythrocytic parasite (trophozoite and schizont) remodel the cytoskeleton and plasma membrane to create cytoadherence knobs as well as nutrient permeation pathways and alter the mechanical stability of the erythrocytes, causing them to stick to blood vessels [2], [3]. This leads to blockage of the microcirculation and results in dysfunction of multiple organs, typically the brain in cerebral malaria [4].

Calcium (Ca^2+^) oscillations are ubiquitous intracellular signals responsible for numerous cellular processes, including excitation–contraction coupling, fertilization, cell growth, transformation, secretion and neuronal signalling [5], [6]. In apicomplexan parasites, such as *Plasmodium*, *Toxoplasma* and *Cryptosporidium*, Ca^2+^-mediated signalling controls various vital functions such as protein secretion, motility, cell invasion and differentiation [7]–[15]. With regard to intracellular Ca^2+^ signalling in *Plasmodium* species, numerous studies have focused on calcium-dependent protein kinases, which are activated downstream of Ca^2+^ release from intracellular Ca^2+^ stores, as an important therapeutic target for antimalarial drug development [8], [9], [12], [15], [16]. Particularly in the blood stage, Ca^2+^ has been considered to be a key regulator of the parasite egress and invasion of erythrocytes [16]–[18]; however, little is known about the role of Ca^2+^ signalling in intraerythrocytic development of *Plasmodium* species.

In this study, we observed the intracellular dynamics of Ca^2+^ throughout the intraerythrocytic stages of the FCR-3 strain of *P.* *falciparum* and found that stage-specific spontaneous Ca^2+^ oscillations which can be blocked by the inositol 1,4,5-trisphosphate (IP_3_) receptor inhibitor 2-aminoethyl diphenylborinate **(**2-APB) occur in the ring form and trophozoite. Examination of the effects of 2-APB on the *in vitro* intraerythrocytic parasite development and electron microscopic observations revealed that blockage of Ca^2+^ oscillations caused severe degeneration and breakdown of successive asexual reproduction in the intraerythrocytic parasites, resulting in death of them. Furthermore, 2-APB showed a similar effect against the chloroquine-resistant K1 strain of *P. falciparum*.

## Results and Discussion

### IP_3_-induced Ca^2+^ Oscillation in *P. falciparum*

**Figure 2:**
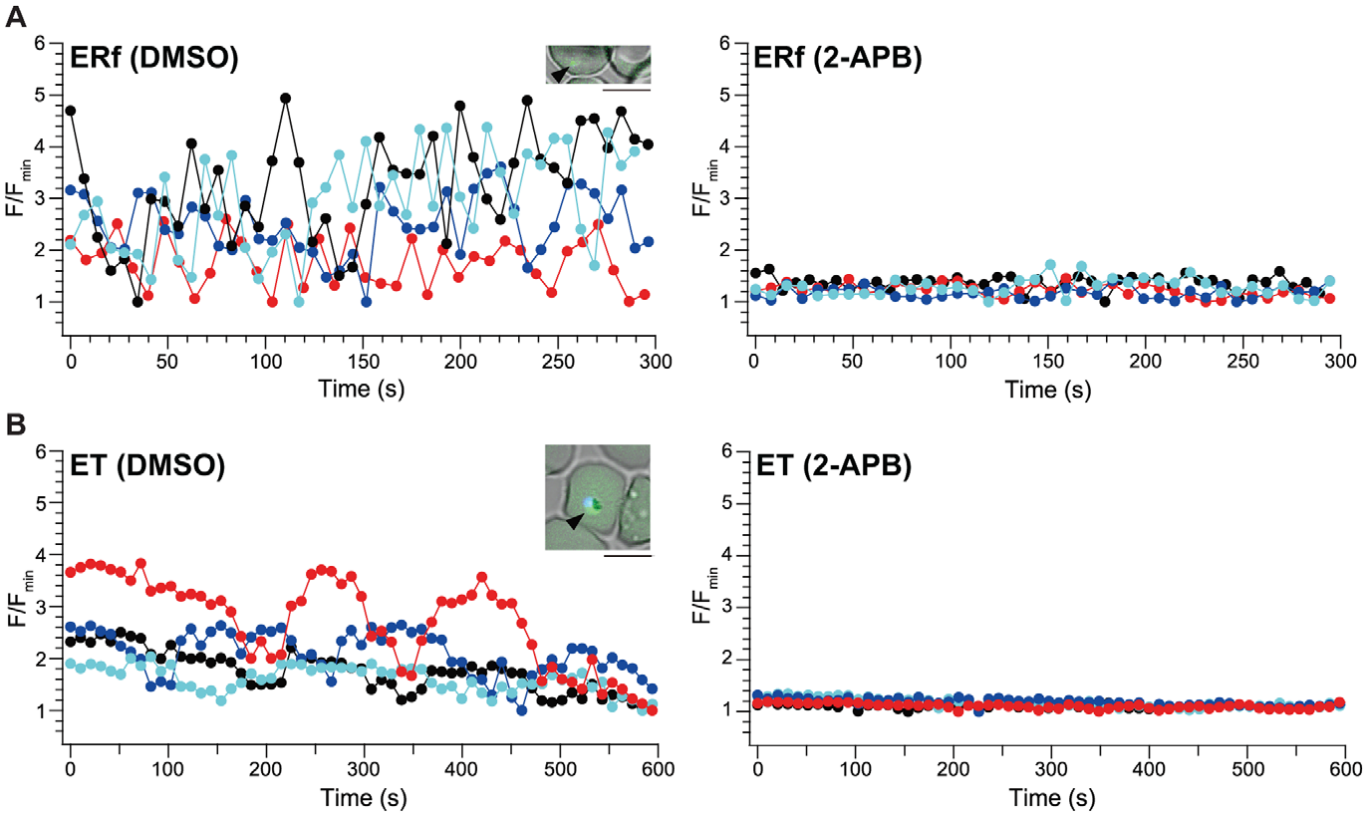
Cytosolic calcium (Ca^2+^) dynamics in the early ring forms (ERf) (A) and early trophozoites (ET) (B) and effects of 2-aminoethyl diphenylborinate (2-APB). Each colour represents cytosolic Ca^2+^ dynamics acquired from individual parasites in the presence (right columns) or absence (left columns) of 100 µM 2-APB. Embedded images in left panels are representative images of Fluo-4-loaded *P. falciparum* during each intraerythrocytic stage (indicated by arrowheads). Scale bars, 5 µm.

Figure 2 and S1 shows fluorescence Ca^2+^ images of each intraerythrocytic developmental stage of the FCR-3 strain: early ring forms (ERf, parasites with smaller cell size than trophozoite without malaria pigment), late ring forms (LRf, parasites with cell size between early ring form and trophozoite without malaria pigment), early trophozoites (ET, parasites with a single nucleolus, malaria pigment and immature food vacuole), late trophozoites (LT, parasites with a single nucleolus and mature food vacuole), schizonts (S, parasites with multiple nuclei) and merozoites (M). Ca^2+^ imaging of parasites was performed in culture chambers at 37°C in an atmosphere of 5% O_2_ and 5% CO_2_, conditions identical to those in conventional *in vitro* parasite culture. The Fluo-4 fluorescence in a parasite cytoplasm (F) was calculated by subtraction of the background fluorescence and normalized to the minimum fluorescence during the imaging period (F_min_). In early ring forms (ERf) and early trophozoites (ET), spontaneous Ca^2+^ oscillations were observed (Fig. 2A and B, left). Dimethyl sulfoxide (DMSO) was used as a solvent control. The frequency of Ca^2+^ oscillations was higher in early ring forms than that in early trophozoites. The subcellular distribution of Fluo-4 in the early trophozoites indicates that free Ca^2+^ were evenly distributed in the cytoplasm (Fig. 2B), whereas in the late trophozoites with mature food vacuole, Ca^2+^ gradient between the digestive food vacuole and cytoplasm, similar to that previously reported [19], [20] was observed being independent of the addition of 2-APB (Fig. S2). 2-APB was a well-established inhibitor of IP_3_ receptor/Ca^2+^ channels developed in our previous study [21], [22] and the blockage of melatonin-induced Ca^2+^ release by 2-APB in *P. falciparum* has been demonstrated [23]. Treatment with 100 µM 2-APB almost completely blocked Ca^2+^ oscillations (Fig. 2A and B, right). On the other hand, in the late ring forms (LRf), late trophozoites (LT), schizonts (S) and merozoites (M), small periodic Ca^2+^ fluctuations were observed, and notable effects of 2-APB were not detected (Fig. S1). To investigate the effects of 2-APB in detail, we performed quantitative analysis of the effect of 2-APB on the amplitude of periodic Ca^2+^ fluctuations in late ring form, late trophozoite, schizont and merozoite stages. The mean amplitude was calculated by subtracting the mean minimal value of F/F_min_ from its mean maximal value. A statistically significant effect of 2-APB was observed in merozoites (*, *P* = 0.0116, two-tailed unpaired *t* test) (Fig. S3). This result supports those of previous studies demonstrating that an increase in cytosolic Ca^2+^ concentration is involved in the regulation of merozoite invasion of the erythrocytes [16]–[18]. In apicomplexan parasites, Ca^2+^ transients and oscillations have been reported to be evoked by several inducers [10], [13], [24], [25]; however, our study is the first to demonstrate spontaneous Ca^2+^ oscillations, i.e. without the addition of exogenous inducers.

Blockage of spontaneous Ca^2+^ oscillations by 2-APB in early ring forms and early trophozoites strongly suggests that the observed Ca^2+^ oscillations are regulated by a putative IP_3_ receptor/Ca^2+^ channel that is activated by IP_3_ binding. In *Plasmodium* the phospholipase C (PLC) pathway is known to be involved in the release of Ca^2+^ from the intracellular Ca^2+^ store, and the effectiveness of U73122, a commonly used PLC inhibitor, has been demonstrated [25]. PLC catalyses the hydrolysis of 1-phosphatidyl-D-myo-inositol-3,4,5-trisphosphate to the second messenger molecules diacylglycerol and IP_3_. As shown in Fig. S4A, after 5 min of pre-treatment with 10µM U73122, Ca^2+^ oscillations in early ring forms (ERf, left) and early trophozoites (ET, right) were almost completely inhibited. In apicomplexan parasites, two intracellular Ca^2+^ stores are known to be involved in Ca^2+^ release: the endoplasmic reticulum (ER) and acidocalcisomes including the food vacuole [26], [27]. To investigate the source of the observed Ca^2+^ oscillations, we depleted Ca^2+^ stores before imaging by using thapsigargin (Tg), a specific inhibitor of sarco/endoplasmic reticulum Ca^2+^–ATPase, and concanamycin A (CMA), a specific inhibitor of vacuolar-type H^+^-ATPase. The effects of these inhibitors have been demonstrated in *Plasmodium* species [28]–[30]. We also confirmed the effectiveness of these compounds in depleting Ca^2+^ by Ca^2+^ imaging in early ring forms and early trophozoites using perfusion experiments (Fig. S4B and C). As shown in Fig. S4D, Ca^2+^ depletion by pre-treatment with 2 µM Tg for 30 min significantly reduced Ca^2+^ oscillations in early ring forms (ERf, left) and early trophozoites (ET, right). In contrast, Ca^2+^ depletion by pre-treatment with 100 nM CMA had no effect on Ca^2+^ oscillations in early ring forms (ERf, left) and early trophozoites (ET, right) (Fig. S4E). In these two stages the digestive food vacuole, which is known to be a Ca^2+^ pool sensitive to both thapsigargin and a vacuolar-type H^+^-ATPase inhibitor, bafilomycin A_1_ [19], is not formed. Taken together, these results indicate that spontaneous IP_3_-induced Ca^2+^ release from a thapsigargin-sensitive Ca^2+^ store, ER occurred in early ring forms and early trophozoites during intraerythrocytic *P. falciparum* development. Next, we investigated the effects of 2-APB on the intraerythrocytic *P. falciparum* development to understand the physiological roles of these spontaneous IP_3_-induced Ca^2+^ oscillations.

**Figure 3:**
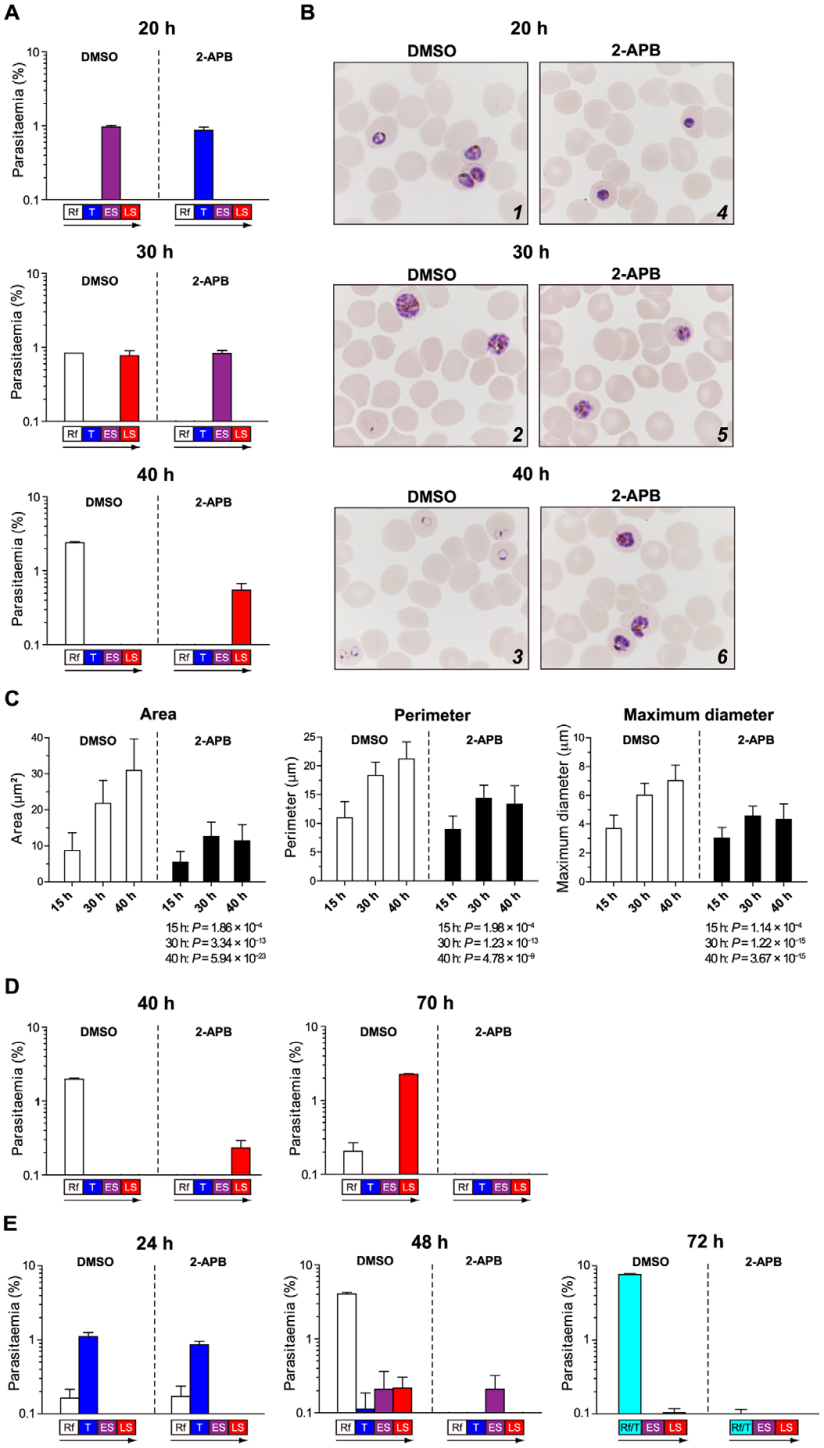
Inhibition of intraerythrocytic *P. falciparum* development by 2-APB. (A) The FCR-3 strain was cultured for 40 h of the intraerythrocytic development cycle. Cultures were terminated at 20, 30 and 40 h of the assay after synchronization, and thin smears of erythrocytes were prepared for parasite counting. Representative results of 3 independent experiments are shown. (B) Morphology of intraerythrocytic parasites cultured with DMSO or 100 µM 2-APB at 20, 30 and 40 h of the assay after synchronization. (C) 100 µM 2-APB significantly decreased the area, perimeter and maximum diameter of intraerythrocytic parasites at 15, 30 and 40 h of the assay after synchronization. Columns and error bars represent the mean + S.D. Fifty parasites were measured at each time point. The *P* values compared with DMSO controls are given below each figure (two-tailed unpaired *t* test with Welch’s correction). (D) Cultures were terminated at 40 and 70 h of the assay after synchronization for parasite counting. Culture medium with DMSO or 2-APB was replaced at 40 h. Representative results of 3 independent experiments are shown. (E) The chloroquine-resistant strain K1 was cultured for 72 h of the intraerythrocytic development cycle. Cultures were terminated at 24, 48 and 72 h of the assay after synchronization, and thin smears of erythrocytes were prepared for parasite counting. Culture medium with DMSO or 2-APB was replaced at 24 and 48 h. Representative results of 3 independent experiments are shown. Parasiaemia of ring form (Rf), trophozoites (T), early schizonts (ES) and late schizonts (LS) is shown as mean + S.D. of 3 independent counts of a single well (A) or 3 wells (D, E). Stages with parasitaemia of less than 0.1% are not shown (A, D and E).

### 2-APB Blocks *P. falciparum* Development

The effects of 2-APB on intraerythrocytic development were investigated using synchronized parasite cultures in the ring form stage, with initial parasitaemia of approximately 1%. Figure 3A shows parasitaemia of each developmental stage at 20, 30 and 40 h of the assay. In the presence of 100 µM 2-APB intraerythrocytic development of the parasites was delayed compared to that in the presence of dimethyl sulphoxide (DMSO). Figure 3B shows the morphology of intraerythrocytic parasites. Parasites cultured with DMSO developed into early schizonts (parasites with fewer than 8 nuclei) at 20 h of the assay (Fig. 3B, panel 1). These schizonts developed into healthy late schizonts (parasites with at least 8 nuclei) and produced ring forms in the next developmental cycle at 30 and 40 h of the assay (Fig. 3B, panels 2 and 3). In contrast, parasites cultured with 2-APB remained at the trophozoite stage (parasites with a single nucleus) with abnormal morphology at 20 h of the assay (Fig. 3B, panel 4). These abnormal trophozoites could develop into early schizonts and late schizonts but exhibited abnormal morphology at 30 and 40 h of the assay (Fig. 3B, panels 5 and 6). To quantitatively examine the effect of 2-APB on intraerythrocytic parasite development, the area, perimeter and maximum diameter of the parasites were analysed at 15, 30 and 40 h of the assay. At 40 h of the assay only schizonts were analysed. At 15, 30 and 40 h of the assay, parasites cultured with 2-APB showed significantly decreased size compared to those cultured with DMSO, indicating that 2-APB delayed intraerythrocytic parasite development (Fig. 3C). Furthermore, analysis of the parasite size revealed that the increases in the 3 parameters were terminated at 30 h after 2-APB treatment, suggesting that the critical time of the effect of 2-APB is approximately 30 h. To investigate the fate of abnormal schizonts at 40 h of the assay in which parasites were cultured with 2-APB (Fig. 3B, panel 6), the assay was prolonged for another 30 h (Fig. 3D). Results at 40 h of the assay confirmed those shown in Fig. 3A. At 70 h parasites cultured with 2-APB developed into a few late schizonts or ring forms with abnormal morphology (1 in 5000–8000 erythrocytes), although those cultured with DMSO developed normally into late schizonts or ring forms in the next developmental stage. On the other hand, parasites could develop normally in 2-APB-pretreated erythrocytes similar to that in cultures with DMSO-pre-treated erythrocytes (Fig. S5). This indicates that the effect of 2-APB is not due to the disruption of erythrocyte physiology, which is significant to intraerythrocytic parasite development and invasion. From these results we conclude that 2-APB directly inhibits intraerythrocytic parasite development by blocking its normal cell cycle, resulting in a failure to maintain the successive developmental stages of asexual blood forms.

**Figure 4:**
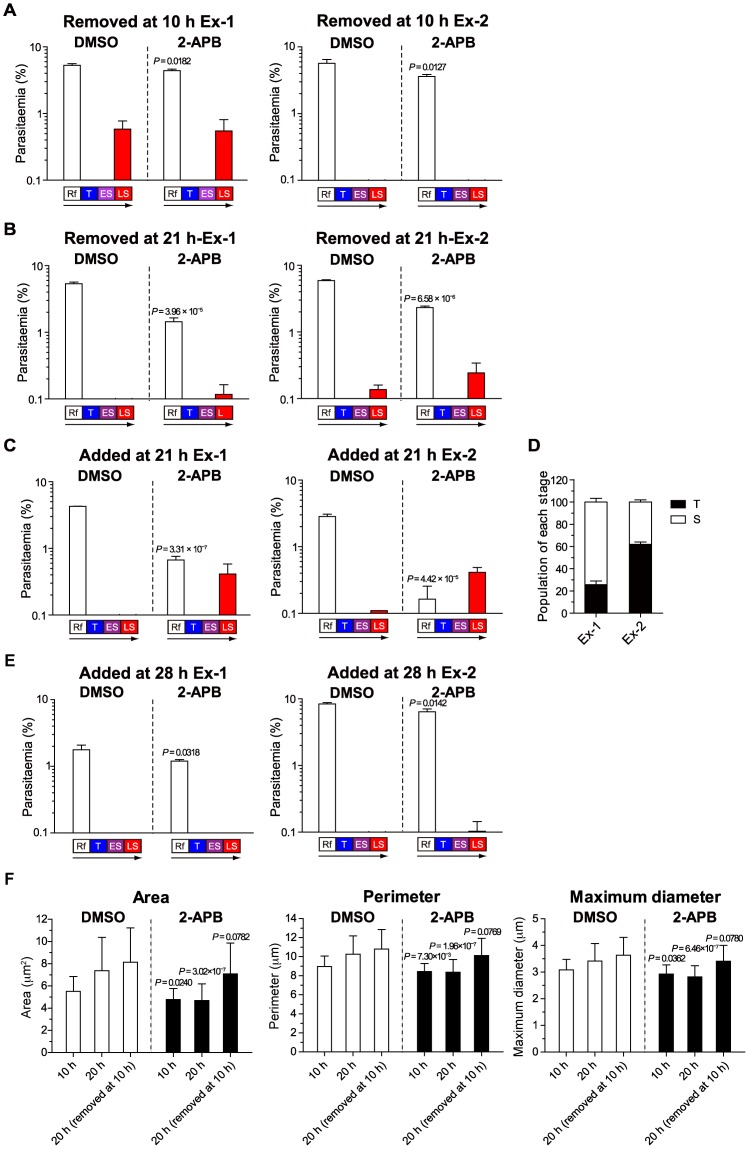
Time window for inhibition of intraerythrocytic *P. falciparum* development by 2-APB. (A) 2-APB was removed at 10 h of the assay. (B) 2-APB was removed at 21 h of the assay. (C**)** 2-APB was added at 21 h of the assay. (D) Percentages of T and S in 2 independent experiments shown in (C) just before 2-APB was added at 21 h of the assay. (E) 2-APB was added at 28 h of the assay. Parasitaemia at 40 h of the assay is shown as mean + S.D. of 3 independent counts of 3 wells. Stages with parasitaemia of less than 0.1% are not shown. The difference in Rf parasitaemia between the DMSO and 2-APB groups was analysed statistically (two-tailed unpaired *t* test ) and *P* values are given in each panel (A, B, C and E). (F) Effects of 100 µM 2-APB on the area, perimeter and maximum diameter of parasites. Three experimental groups were tested as follows. 2-APB was added at the beginning of the assay during synchronization, and cell size was analysed at 10 or 20 h of the assay. 2-APB was added at the beginning of the assay, removed at 10 h of the assay and cell size was measured at 20 h of the assay. Fifty parasites were measured in each experimental group. *P* values compared with DMSO controls are given in each panel (two-tailed unpaired *t* test with Welch’s correction).

### 2-APB Acts on a Chloroquine-resistant Strain

Next, we examined the effect of 2-APB on the intraerythrocytic development of the chloroquine-resistant strain K1 of *P. falciparum* using synchronized parasite cultures in the ring form stage with initial parasitaemia of approximately 2%. At 24 h of the assay a tendency towards decreasing trophozoite parasitaemia was reproducibly observed in parasites cultured with 100 µM 2-APB (Fig. 3E, 24 h). The slight inhibitory effect of 2-APB at 24 h of the assay was confirmed by measuring the area, perimeter and maximum diameter of the parasites (Fig. S6). At 48 h of the assay, intraerythrocytic parasite development in the presence 2-APB was delayed compared to that in the presence of DMSO, similar to that observed in the FCR-3 strain (Fig. 3E, 48 h). A further 24 h of the assay revealed that the number of infected erythrocytes in the presence of 2-APB was much lesser than that in the presence of DMSO, in which there was a high level of parasitaemia (Fig. 3E, 72 h).

**Figure 5:**
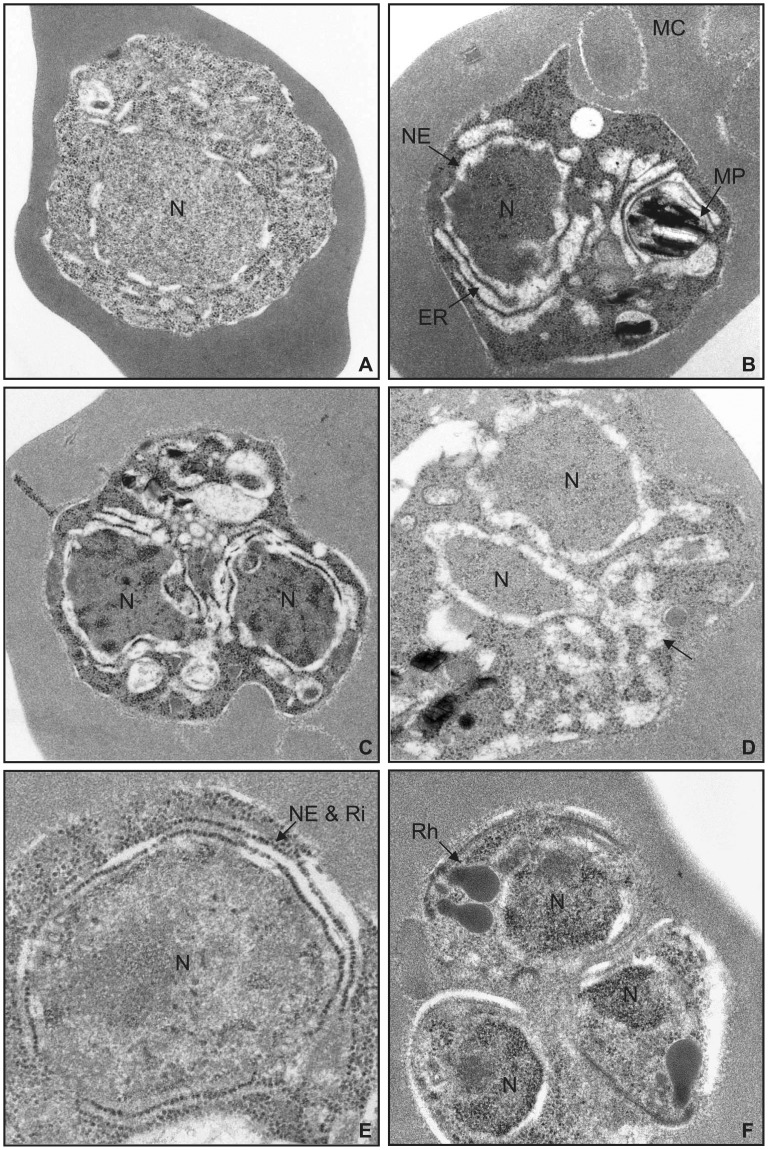
Electron micrographs of parasites treated with 2-APB. (A) After 30 h of DMSO administration (control group; original magnification, ×30,000). (B) After 30 h of 100 µM 2-APB administration (original magnification, ×30,000). (C) After 30 h of 100 µM 2-APB administration (original magnification, ×30,000). (D) After 30 h of 100 µM 2-APB administration. Reticular ER structure is observed (arrow) (original magnification, ×50,000). (E) After 40 h of 100 µM 2-APB administration. The nuclear envelope (NE) surrounded by ribosomal granules (Ri) is observed (arrow) (original magnification, ×50,000). (F) After 40 h of 100 µM 2-APB administration. Formation of rhoptries (Rh) and other micro-organelles is observed (original magnification, ×50,000). N, nucleus; MP, malaria pigment; MC, Maurer’s cleft.

**Figure 6:**
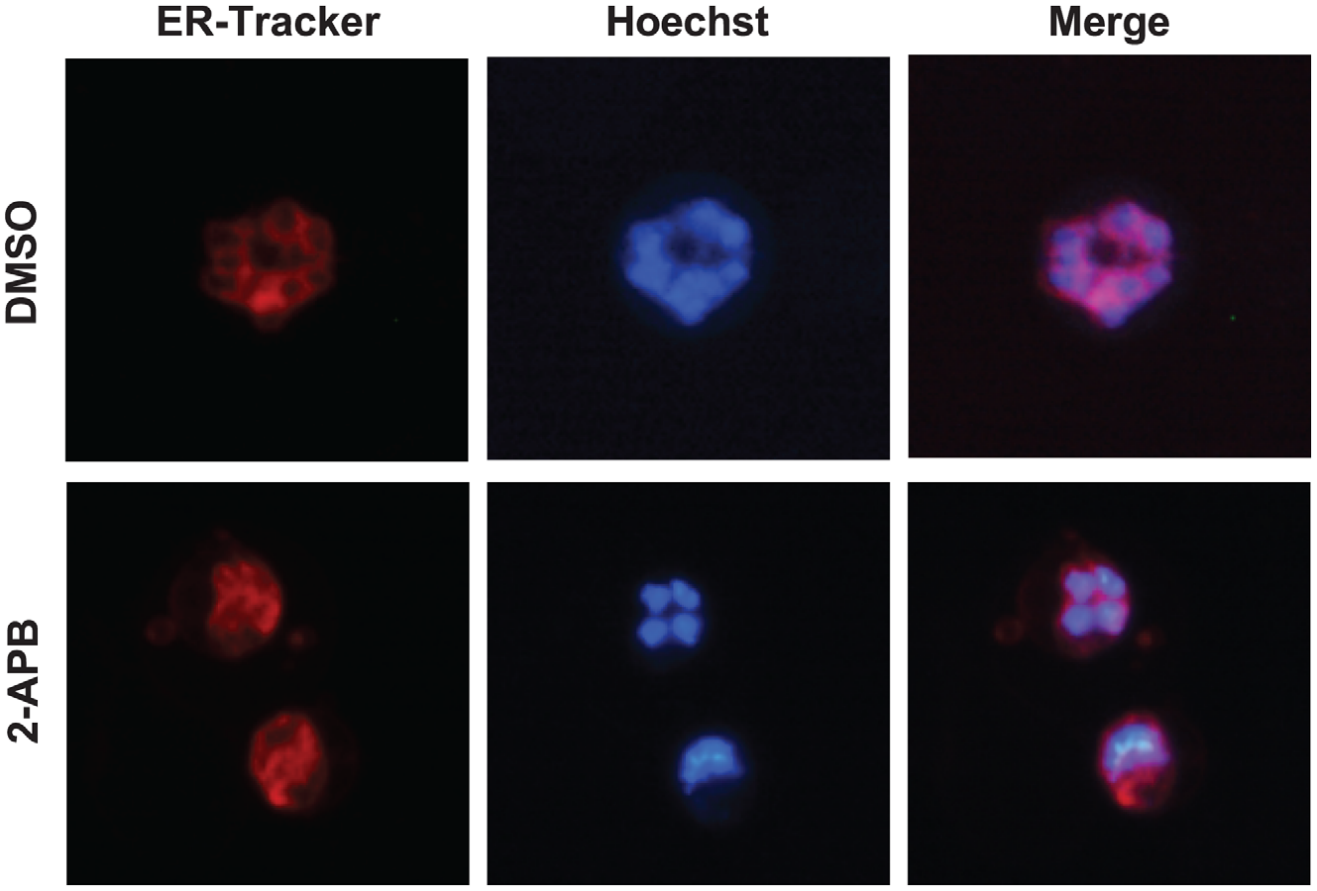
Effect of 2-APB on ER structure. The parasite nucleus and ER were stained simultaneously with Hoechst 33342 (blue) and ER-Tracker (red) after 30 h of DMSO (upper panels) or 2-APB (lower panels) administration. Merged images are shown in the right columns (Merge).

### Trophozoites are the Main Target of 2-APB

To investigate the stages of intraerythrocytic development that 2-APB may block, 2-APB was removed from or added to cultures at different time intervals and parasitaemia was determined after 40 h of the assay for each developmental stage. Two independent results are shown in Fig. 4A-E. When 2-APB was removed from culture at the ring form stage (10 h of the assay) (Fig. 4A), parasites present at 40 h of the assay developed into schizonts with normal morphology, but ring form parasitaemia in the next developmental cycle was significantly decreased. On the other hand, when 2-APB was removed between trophozoite to early schizont stages (21 h of the assay) (Fig. 4B), parasites present at 40 h of the assay developed into schizonts with either normal or abnormal morphology, and the effect of 2-APB in decreasing ring form parasitaemia in the next developmental cycle was greater than that shown in Fig. 4A. When 2-APB was added at the same time (21 h of the assay) (Fig. 4C), parasites present at 40 h of the assay developed into schizonts with abnormal morphology and ring form parasitaemia in the next developmental cycle was significantly decreased. These results indicate that the time at which 2-APB is most effective in blocking intraerythrocytic parasite development is between the trophozoite to early schizont stages. This is supported by the observation that a higher percentage of trophozoites at the time of addition of 2-APB resulted in greater effects on ring form parasitaemia (Fig. 4D). Furthermore, when 2-APB was added at a later stage (late schizont stage, 28 h of the assay), the parasites were able to develop into fully mature schizonts with segmented merozoites and produce ring forms like those observed in the control culture (Fig. 3B); however, ring form parasitaemia at 40 h of the assay was significantly decreased (Fig. 4E). This result suggests that 2-APB affects late schizont maturation and erythrocyte egress and/or invasion of merozoites. Finally, to further investigate the effect of 2-APB on the ring form stage, parasite size was measured at 10 and 20 h of the assay (Fig. 4F). In parasites cultured with DMSO, increases in area, perimeter and maximum diameter at 20 h of the assay were higher than those observed at 10 h of the assay as expected from the results in Fig. 3C. A similar result was obtained when DMSO was removed at 10 h of the assay. In contrast, in parasites cultured with 2-APB at 10 and 20 h of the assay, all 3 parameters were significantly smaller than those in parasites cultured with DMSO; however, when 2-APB was removed at 10 h of the assay, all 3 parameters were slightly smaller than those in parasites cultured with DMSO but recovered to a level at which no statistically significant difference was detected. From these results we propose 2 possibilities to explain the finding that removal of 2-APB at 10 h of the assay caused a significant decrease in ring form parasitaemia at 40 h of the assay (Fig. 4A). (1) The growth of trophozoites that were exposed to 2-APB before 10 h of the assay was inhibited. and (2) a proportion of ring forms did not recover and resume normal development. Together with results of Ca^2+^ imaging experiments (Fig. 2), the inhibitory effects of 2-APB on Ca^2+^ oscillations and intraerythrocytic parasite development in the blood stage are summarized in Fig. S7. Ca^2+^ oscillations observed in early ring forms and early trophozoites reduced on treatment with 2-APB (Fig. 2A and B), but treatment at the ring form stage showed a significantly weaker effect on intraerythrocytic development than that between trophozoite to schizont stages. Considering the reversible effect of 2-APB on the early ring form stage (Fig. 4F), we conclude that the lethal effect of 2-APB on intraerythrocytic parasite development was caused mainly by the blockage of Ca^2+^ oscillations in the early trophozoite stage. The importance of Ca^2+^-mediated signals for merozoites invasion of erythrocytes has been reported previously [17], [18], and hence, the results in Fig. 4E suggest that IP_3_-induced periodic Ca^2+^ fluctuations in the merozoites stage has an important role in parasite invasion. Moreover, the weak effect of 2-APB on strain K1 at 24 h of the assay (Fig. 3E) may be attributable to the fact that most of the K1 parasites did not reach the stage (trophozoite to early schizont stage) at which 2-APB had a lethal effect in the FCR-3 strain (Fig. 4). However, it remains possible that the effectiveness of 2-APB in the ring form stage of strain K1 differs from its effectiveness in the FCR-3 strain.

### Severe Parasite Degeneration Caused by 2-APB

Ultrastructural changes induced by 2-APB were observed by transmission electron microscopy. As shown in Fig. 5A and Fig. S8, parasites cultured with DMSO at 30 h of the assay maintained a normal structure. In contrast, highly dense chromatin masses in the nucleus and highly dense degeneration were consistently observed 30 h after the assay (Fig. 5B and C). The formation of Maurer’s cleft and malaria pigment in the food vacuoles suggests that degeneration induced by 2-APB occurred after intraerythrocytic development to some extent (Fig. 5B). In *Plasmodium* species, the nuclear envelope is considered the main ER compartment [31]–[34]. We confirmed that the ER-Tracker signals surrounded the nuclei of parasites stained blue with Hoechst 33342 and cultured with DMSO, whereas in parasites cultured with 2-APB the ER-Tracker signals became broad and extended to the cytosol (Fig. 6). Similarly, in electron micrographs parasites cultured with 2-APB characteristically showed a dilated nuclear envelope and ER (Fig. 5B and C) and the dilated nuclear envelopes connected with the dilated ER, forming a reticular ER structure (Figs 5D and 6). In a previous report, a nucleus surrounded by rough ER in a late schizont with the nuclear envelope bearing numerous ribosomal granules was observed by electron microscopy [35] and similar electron micrographs were obtained with parasites cultured with DMSO in this study (Fig. S8B). Interestingly, parasites cultured with 2-APB frequently exhibited an increased number of ribosomal granules, distributed at even intervals in a line along the dilated nuclear envelope (Fig. 5E). Similar morphological alterations in the nuclear envelope and rough ER were also observed in some parasites exposed to artemisinin [36], which is known to inhibit the sarco/endoplasmic reticulum Ca^2+^-ATPase orthologue (PfATP6) of *P. falciparum* [37], suggesting that these ultrastructural changes are commonly induced by interference with Ca^2+^ signalling. Dilatation of the nuclear envelope and ER is possibly among the features of cell degeneration, but it may also compensate for disrupted Ca^2+^ signalling, since ER is a multifunctional and highly dynamic organelle, undergoing constant movement and reorganization depending on cellular conditions [38], [39]. Although most of the parasites cultured with 2-APB showed severe degeneration (Fig. 5B and C), schizonts in which merozoites with normal micro-organelles were formed were also present (Fig. 5F). At 40 h of the assay, the number of merozoites formed in each schizont of parasites cultured with 2-APB was significantly smaller than that formed in parasites cultured with DMSO (Fig. S9A and B), suggesting that parasite development was inhibited by 2-APB even in parasites in which merozoites were formed normally.

### Conclusive Remarks

In higher eukaryotes, disrupted intracellular Ca^2+^ signalling and accumulation of aberrant proteins is known to cause ER stress, a hallmark of cell death that is associated with many neurodegenerative diseases [40]–[43]. However, very little is known about the biological significance and molecular composition of Ca^2+^ signaling systems in unicellular organisms. In this study, we clearly showed the stage-specific spontaneous Ca^2+^ oscillations in the intraerythrocytic stages of *P. falciparum*, a unicellular eukaryote, without any exogenous extracellular stimulation. We further demonstrated that the blockage of these Ca^2+^ oscillations by 2-APB caused severe cellular degeneration resulting to the death of the parasites. The half maximal inhibitory concentration (IC_50_) value of 2-APB for inhibition of IP_3_-induced Ca^2+^ release in mammalian cells was ∼40 µM [21]. Thus, a high concentration (100 µM) 2-APB was used in this study. Such a high dose 2-APB possibly exerts pleiotropic effects on both host cells and parasites. However, our findings in this study strongly support the idea that the severe inhibitory effect of 2-APB on the intraerythrocytic development of the parasites is primarily due to the specific blockage of Ca^2+^ oscillations of the early trophozoites. First, the normal development of the parasites was observed in 2-APB-pretreated erythrocytes (Fig. S5). Secondly, the critically effective time window of 2-APB on the intraerythrocytic development of the parasites was coincident with the early trophozoite stage in which spontaneous Ca^2+^ oscillation was observed (Fig. 2, 4 and S7). Thirdly, the delayed development of the parasites was recovered when 2-APB was removed at 10 h of the assay (Fig. 4F). Lastly, 100 µM 2-APB did not disrupt Ca^2+^ gradient between the food vacuole and cytoplasm unlike a µM order of chloroquine treatment permeabilized the food vacuole membrane, resulting in the cell death [20]. Interestingly, a much higher contribution of the Ca^2+^ oscillations in trophozoite stage to the intraerythrocytic development of the parasites was observed than that in the ring form stage. This result indicates that the Ca^2+^ oscillation observed in ring form stage has a different physiological role from that in the trophozoite stage. In *Plasmodium* species, gametocytogenesis delivers the sexual-stage of the parasite known as gametocyte involved in the transmission from the mammalian host to the mosquito. Gametocyte development can be induced by host factors or drug treatment, and of which signal transduction pathways are involved in this process [44]. There is consistent evidence that phorbol ester inducing pathways and cyclic AMP (cAMP) signalling pathway are involved in triggering gametocytogenesis of *P. falciparum* [45]–[49] and interplay between cAMP and Ca^2+^ as second messengers was also reported [24]. Taken together, our results suggest the possibility that Ca^2+^ oscillations during ring form stage might be involved in triggering gametocytogenesis, rather than in maintaining asexual erythrocytic cycle.

During the erythrocytic cycle, *Plasmodium* species repeat the drastic morphological and functional changes; invasion, feeding, multiplication, secretion and structural modification of the parasite-infected erythrocytes. Our study clearly indicated that Ca^2+^ signaling plays a pivotal role for cell growth and differentiation of *P. falciparum*, suggesting that it could be a useful experimental model organism for understanding fundamental roles and mechanisms of Ca^2+^ signaling conserved from unicellular organisms to humans. In clinical aspects, the mature-stage of *P. falciparum* modifies the surface structure of the host erythrocytes. The parasite-infected erythrocytes adhere to endothelium and sequester the microvasculature of several organs and block the blood circulation. This pathology called “sequestration” causes severe symptoms including coma, acute respiratory distress, kidney failure and death in humans. Thus, consequences of malaria are closely associated with intraerythrocytic *P. falciparum* development. Although functional evidence for IP_3_-induced Ca^2+^ release has been reported in *Plasmodium* species [50], [51], little attention has been paid to the molecules upstream of Ca^2+^ release from intracellular Ca^2+^ stores due to the lack of molecular identities for them. An IP_3_R gene, as defined in metazoans, has not been identified in the *Plasmodium* genome. This apparent absence could be due to the lack of homology with IP_3_Rs in metazoans as in plants [26], [52]. Our results increase the possibility that the identification of molecules responsible for generating Ca^2+^ oscillations including IP_3_R will provide promising targets for the development of novel antimalarial drugs.

## Materials and Methods

### *P. falciparum* Culture

The FCR-3 and K1 strains of *P. falciparum* were cultured using the modified method of Trager and Jensen [53] in RPMI medium (Invitrogen/Gibco) supplemented with 0.5% Alubumax I (Invitrogen), 25 mM HEPES, 24 mM sodium bicarbonate, 0.5 g/L L-glutamine, 50 mg/L hypoxanthine, 25 µg/mL gentamicin (Sigma) and human erythrocytes (from a healthy Japanese volunteer) at a haematocrit of 5% was used for culture. Growth synchronization was achieved with 5% *D*-sorbitol [54].

### Fluorescence Ca^2+^ Imaging

A culture of infected erythrocytes (0.5 mL; 5 × 10^8^ erythrocytes/mL) was diluted 10-fold with BSA(−) medium for Ca^2+^ imaging (RPMI 1640 medium without phenol red; Invitrogen/Gibco) supplemented with 25 mM HEPES, 24 mM sodium bicarbonate, 0.5 g/L L-glutamine and 50 mg/L hypoxanthine. Cultures of erythrocytes (1 mL) were collected by centrifugation (1000 *g* for 5 min at room temperature) and resuspended in 350 µL BSA(−) medium. Loading Solution was prepared for staining nuclei with Hoechst 33342 and loading of Fluo-4-AM [0.1 mg/mL Hoechst 33342 (Dojindo), 20–100 µM Fluo-4-AM (Invitrogen/Molecular Probes), 100-fold dilution of PowerLoad (Invitrogen) in BSA(−) medium]. A suspension of erythrocytes (350 µL) was mixed with 150 µL of Loading Solution and shaken at 200 rpm for 30–60 min at 37°C. Erythrocytes were then washed once with 10 mL BSA(−) medium (1000 *g* for 5 min at room temperature) and resuspended in 600 µL of BSA(+) medium [BSA(−) medium supplemented with 0.5% Albumax I and 25 µg/mL gentamicin (Sigma)]. A suspension of erythrocytes (100 µL) was inoculated in a 35 mm glass-bottomed dish (MatTek) that had been coated with 1 mg/mL poly-L-lysine before use. After 30 min of incubation in an O_2_/CO_2_ incubator, suspended erythrocytes were removed by gentle washing with BSA(+) medium. The glass-bottomed dish was then placed in the culture chamber in which O_2_, CO_2_, temperature and humidity were maintained under conditions used for conventional *in vitro* parasite culture.

Sequential time-lapse imaging of Hoechst 33342, Fluo-4-AM and transparent images was performed using the Leica confocal microscope system (Leica TCS SP5; Leica Microsystems) with a 63× (N.A.1.42) oil immersion objective lens and excitation at 410 nm (diode laser) for Hoechst 33342 and 488 nm (Argon laser) for Fluo-4-AM and transparent images. Emissions were collected using the true spectral detection method developed by Leica Microsystems. Images were captured every 5–15 s for 300–600 s. Specific Fluo-4 fluorescence in a parasite (F) was calculated by subtraction of background fluorescence and normalized to minimum fluorescence during the imaging period (F_min_).

### Inhibition of *P. falciparum* Development by 2-APB

The 2-APB concentration used was determined by preliminary experiments with a variety of concentrations ranging from 1 to 150 µM (data not shown). Effects of 2-APB on the intraerythrocytic parasite development were assayed using parasite cultures in the ring form stage with initial parasitaemia of approximately 1%–2%. Cultures (500 µl) were placed in each well of a tissue culture plate (24-well flat-bottomed; Corning). 2-APB was dissolved in DMSO (Hybri-Max®, Sigma) at 10 mM. Stock solutions were diluted with PPMI medium and added to each well of the culture plate to give a defined concentration. DMSO diluted with medium served as control. A drop of cultured erythrocytes was smeared on a glass slide and stained with Giemsa. The number of parasite-infected erythrocytes in 2000 erythrocytes was counted and defined as the level of parasitaemia.

### Parasite Size Calculation and Transmission Electron Microscopy

Giemsa-stained smears were observed under the Nikon Eclipse 80i microscope (Nikon), photographed using the Nikon DXM 1200F camera and uploaded on a personal computer using digital photo manager software (ACT-1; Nikon). To measure parasite size, we randomly selected 50 parasites and manually delineated areas containing parasites with lines on the screen. The area, perimeter and maximum diameter of the parasites were calculated by WinROOF software package Ver.5.8.1 (Mitani, Japan).

### Transmission Electron Microscopy

Transmission electron microscopy was performed as previously described [55]. Specimens for transmission electron microscopy were fixed for 2 h in 2.5% (v/v) glutaraldehyde buffered with 0.1 M phosphate buffer, pH 7.4, at 4°C. They were postfixed in 1% (w/v) osmium tetroxide for 1 h. Fixed specimens were dehydrated in ascending concentrations of ethanol followed by propylene oxide for 15 min, and embedded in Epon 812. The blocks were cut with an ultramicrotome (Porter-Blim MT-2; Ivan Sorvall) with a diamond knife (Diatome). The sections were mounted on 200-mesh copper grids and stained with uranyl acetate and lead citrate, and examined under the JEOL JEM-1011 transmission electron microscope.

### Nucleus and ER Staining

The nucleus and ER of the parasites was stained with Hoechst 33342 and ER-Tracker Red (Invitrogen). Staining with Hoechst 33342 was performed as described above for fluorescence Ca^2+^ imaging. ER-Tracker Red was then added at a final concentration of 0.5 µM to the erythrocyte suspension and shaken at 200 rpm for 30 min at 37°C. Erythrocytes were then washed once and resuspended in RPMI1640 medium without phenol red and observed under fluorescence microscopy (Leica DM 2500).

### Ethics Statement

The human erythrocytes stock for the parasite culture was provided by the Hokkaido Kushiro Red Cross Blood Centre under the ethical guidelines for the blood products and obtained according to their acquisition guidelines. Written informed consent from the donor of the human erythrocytes was obtained. This study was done without a regular review from ethics committees of the Obihiro University of Agriculture and Veterinary medicine, where the parasite culture was made, because the erythrocytes stock was provided as blood product from the Red Cross after completing their ethical review for the experiment.

## Supporting Information

Figure S1 **Cytosolic calcium (Ca^2+^) dynamics during intraerythrocytic** ***P. falciparum*** **development and effects of 2-aminoethyl diphenylborinate (2-APB).** (A–D) Dimethyl sulfoxide (DMSO) was used as a solvent control. Each colour represents cytosolic Ca^2+^ dynamics acquired from individual parasites of late ring forms (LRf), late trophozoites (LT), schizonts (S) and merozoites (M) in the presence (right columns) or absence (left columns) of 100 µM 2-APB. Embedded images in left panels are representative images of Fluo-4-loaded *P. falciparum* during each intraerythrocytic stage (indicated by arrowheads). Scale bars, 5 µm.
(TIF)

Figure S2 **Subcellular Ca^2+^ distribution in late trophozoite treated with 100 µM 2-APB.** The parasite Ca^2+^ and nucleus and were stained simultaneously with Fluo-4 (green) and Hoechst 33342 (blue). Merged images are shown in the right column (Merge). Scale bars, 5 µm.
(TIF)

Figure S3 **Effects of 2-aminoethyl diphenylborinate (2-APB) on mean amplitude of periodic calcium (Ca^2+^) fluctuations.** A significant effect of 100 µM 2-APB (*, *P* = 0.0116, two-tailed unpaired *t* test) was detected only in merozoites. LRf, late ring forms; LT, late trophozoites; S, schizonts; M, merozoites.
(TIF)

Figure S4 **Effects of U73122, thapsigargin (Tg) and concanamycin A (CMA) on Ca^2+^ oscillations in early ring forms (ERf) and early trophozoites (ET).** (A) Spontaneous Ca^2+^ oscillations observed in ERf (left) and ET (right) disappeared after 5 min of pre-treatment with 10 µM U73122. (B, C) Erythrocytes were prepared in a manner identical to that for Ca^2+^ imaging in the culture chamber. Ca^2+^ imaging was initiated under the same conditions as those used for perfusion with normal culture medium. Replacement of perfusion medium with medium containing 2 µM thapsigargin (Tg) (B) or 100 nM concanamycin A (CMA) (C) induced an increase in Ca^2+^ concentration in ERf (open circles) and ET (closed squares). Black horizontal bars represent the period of perfusion with test compounds. (D) Ca^2+^ depletion induced by 30 min of pre-treatment with 2 µM Tg significantly diminished Ca^2+^ oscillations in ERf (left) and ET (right). (E) Ca^2+^ depletion induced by 30 min of pre-treatment with 100 nM CMA had no effect on Ca^2+^ oscillations in ERf (left) and ET (right).
(TIF)

Figure S5 **Pre-treatment of erythrocytes with 2-APB did not inhibit intraerythrocytic development of** ***P. falciparum*** **.** To evaluate the effects of 2-APB on host cells, erythrocytes were pre-treated with 100 µM 2-APB for 1 h at 37°C, washed with RPMI medium and resuspended at a haematocrit of 5% in complete culture medium. Cultures of late schizonts (LS; 0.5% parasitaemia) were diluted four times with pre-treated erythrocytes, and the culture was continued. Cultures (three wells per experimental group) were terminated at 40 h of the assay, and thin smears of erythrocytes were prepared for parasite counting. Parasitaemia with ring forms did not differ significantly between dimethyl sulfoxide (DMSO)- and 2-APB-pre-treated groups (*P* = 0.408, two-tailed unpaired *t* test). Parasitaemia is shown as mean + S.D. of three independent counts of three wells. Stages with parasitaemia of less than 0.1% are not shown.
(TIF)

Figure S6 **Effect of 2-APB on the area, perimeter and maximum diameter of the chloroquine-resistant strain K1.** No statistically significant difference was observed in the area, perimeter and maximum diameter of intraerythrocytic parasites between DMSO- and 100 µ M 2-APB-cultured groups after 24 h of the assay, but the 3 parameters showed a tendency to decrease. Error bars represent mean + S.D. (n = 50). *P* values are given in each panel (two-tailed unpaired *t* test).
(TIF)

Figure S7 **Timetable of the effects of 2-APB on Ca^2+^ dynamics and parasite development in the blood stage of** ***P. falciparum*** **.** Images show Giemsa-stained parasites. Blue bars above the parasite images indicate stages of the parasites in which spontaneous Ca^2+^ oscillations or small periodic Ca^2+^ fluctuations were observed. Red lines represent the period of 2-APB treatment. Red circles indicate the initiation of 2-APB treatment. Black, dark and light grey boxes show the extent of the effect of 2-APB during each sampling period: black, severe effect with developmental delay and abnormal morphology; dark grey, weaker effect with developmental delay and abnormal morphology than that when parasites were exposed to 2-APB for 40 h; light grey, slight effect with developmental delay or abnormal morphology compared to that when parasites were exposed to 2-APB for 40 h.
(TIF)

Figure S8 **Electron micrographs of parasites in DMSO control culture.** (A) At 30 h of the assay (original magnification, ×30,000). (B) Higher magnification image of the part marked with an asterisk in (A) (original magnification, ×80,000). (C) At 30 h of the assay (original magnification, ×20,000). (D) Higher magnification image of the part marked with an asterisk in (C) (original magnification, ×80,000). N, nucleus; NE, nuclear envelope; Ri, ribosome; Rh, rhoptry.
(TIF)

Figure S9 **Effects of 2-APB on the number of merozoites in each schizont.** (A) Box plot of the number of merozoites (M) formed in each schizont (S) after 40 h of culture with DMSO (white boxes) or 2-APB (light grey boxes). The central rectangle spans the first quartile to the third quartile. The segment inside the rectangle shows the median and whiskers above and below the box show the minimum and maximum. 2-APB significantly decreased the number of M per S (two-tailed unpaired *t* test with Welch’s correction). (B) Frequency distribution of the raw data shown in (A).
(TIF)
