# Supplementary figures and images for: Blockage of Spontaneous Ca2+ Oscillation Causes Cell Death in Intraerythrocitic Plasmodium falciparum
Source: PLoS One. 2012 Jul 6;7(7):e39499. doi: 10.1371/journal.pone.0039499 (PMC3391199; doi:10.1371/journal.pone.0039499)

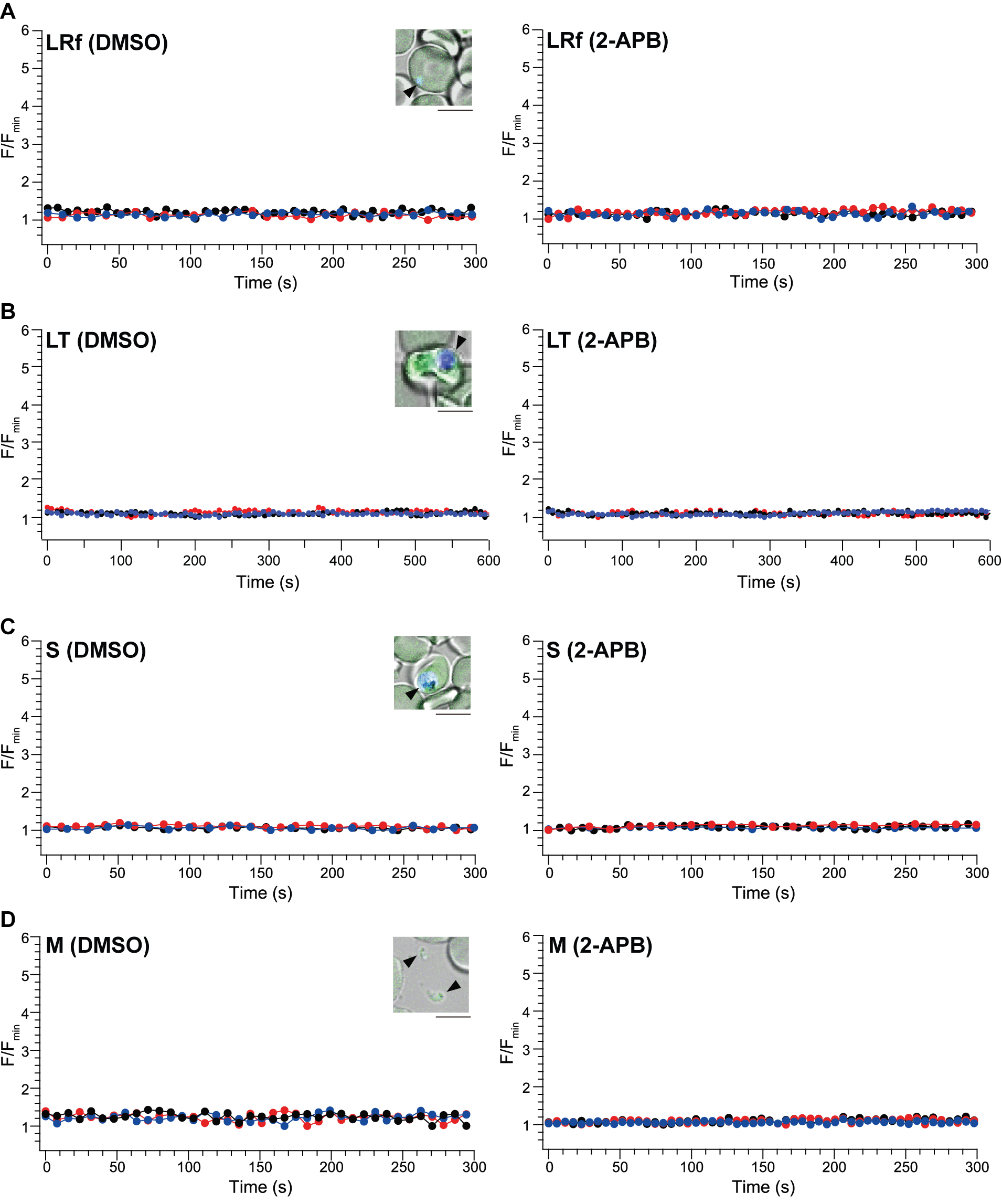

Supplement: Figure S1 — Cytosolic calcium (Ca2+) dynamics during intraerythrocytic P. falciparum development and effects of 2-aminoethyl diphenylborinate (2-APB). (A–D) Dimethyl sulfoxide (DMSO) was used as a solvent control. Each colour represents cytosolic Ca2+ dynamics acquired from individual parasites of late ring forms (LRf), late trophozoites (LT), schizonts (S) and merozoites (M) in the presence (right columns) or absence (left columns) of 100 µM 2-APB. Embedded images in left panels are representative images of Fluo-4-loaded P. falciparum during each intraerythrocytic stage (indicated by arrowheads). Scale bars, 5 µm. (TIF) [file pone.0039499.s001.tif]

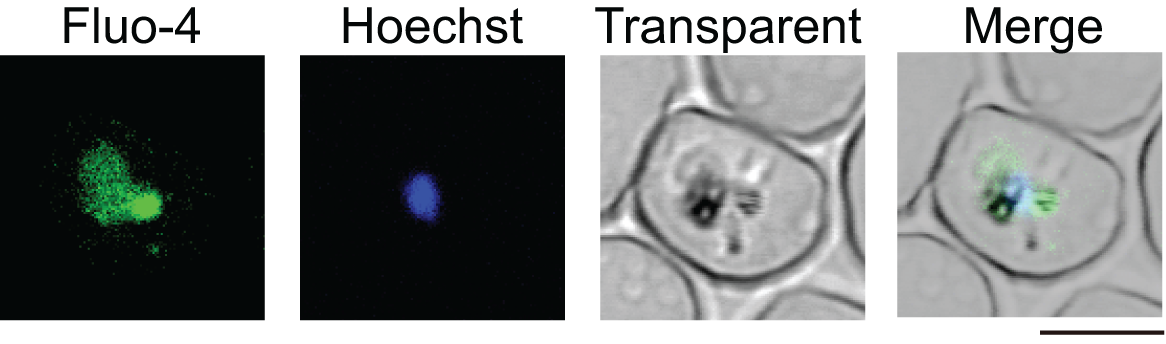

Supplement: Figure S2 — Subcellular Ca2+ distribution in late trophozoite treated with 100 µM 2-APB. The parasite Ca2+ and nucleus and were stained simultaneously with Fluo-4 (green) and Hoechst 33342 (blue). Merged images are shown in the right column (Merge). Scale bars, 5 µm. (TIF) [file pone.0039499.s002.tif]

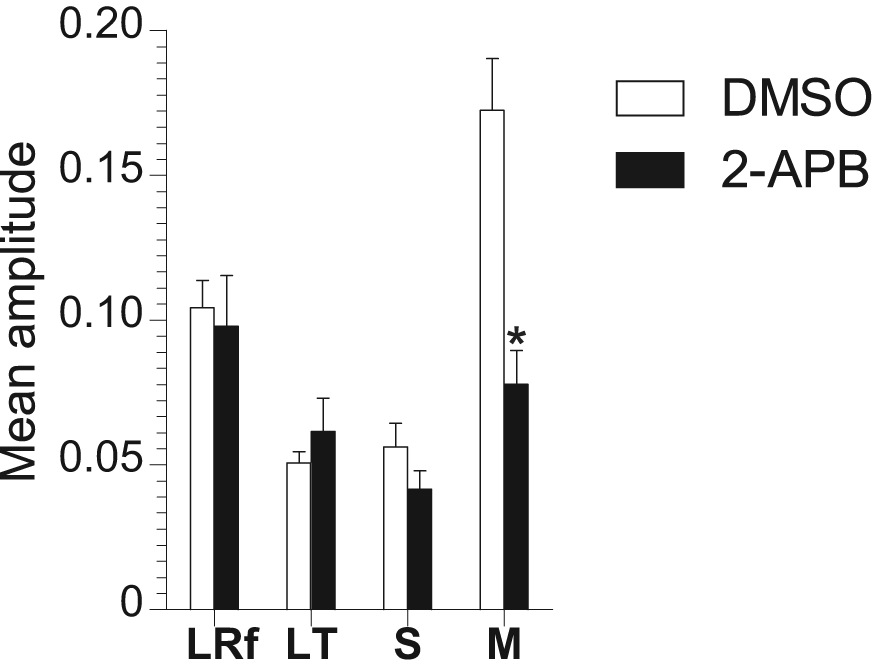

Supplement: Figure S3 — Effects of 2-aminoethyl diphenylborinate (2-APB) on mean amplitude of periodic calcium (Ca2+) fluctuations. A significant effect of 100 µM 2-APB (*, P = 0.0116, two-tailed unpaired t test) was detected only in merozoites. LRf, late ring forms; LT, late trophozoites; S, schizonts; M, merozoites. (TIF) [file pone.0039499.s003.tif]

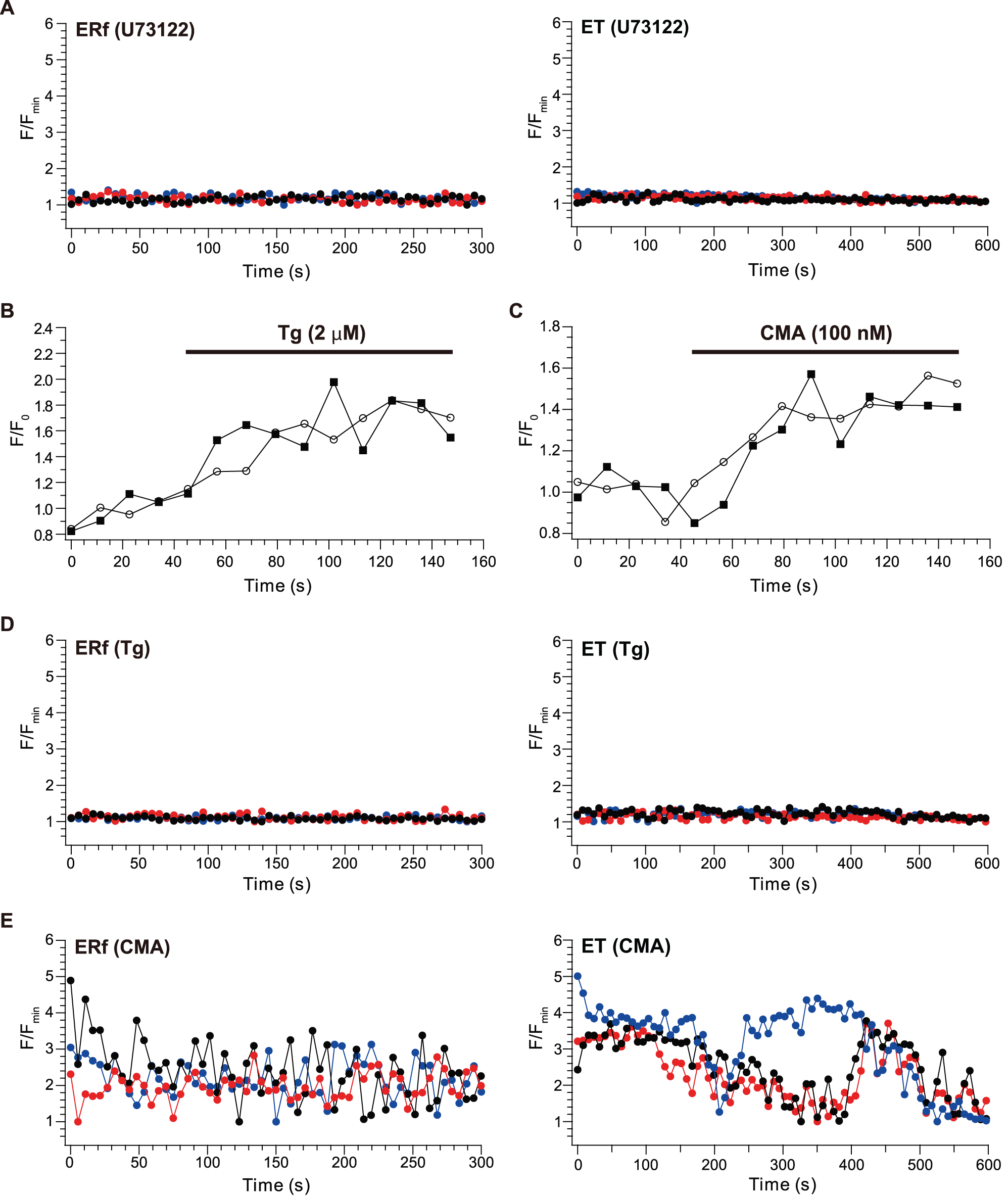

Supplement: Figure S4 — Effects of U73122, thapsigargin (Tg) and concanamycin A (CMA) on Ca2+ oscillations in early ring forms (ERf) and early trophozoites (ET). (A) Spontaneous Ca2+ oscillations observed in ERf (left) and ET (right) disappeared after 5 min of pre-treatment with 10 µM U73122. (B, C) Erythrocytes were prepared in a manner identical to that for Ca2+ imaging in the culture chamber. Ca2+ imaging was initiated under the same conditions as those used for perfusion with normal culture medium. Replacement of perfusion medium with medium containing 2 µM thapsigargin (Tg) (B) or 100 nM concanamycin A (CMA) (C) induced an increase in Ca2+ concentration in ERf (open circles) and ET (closed squares). Black horizontal bars represent the period of perfusion with test compounds. (D) Ca2+ depletion induced by 30 min of pre-treatment with 2 µM Tg significantly diminished Ca2+ oscillations in ERf (left) and ET (right). (E) Ca2+ depletion induced by 30 min of pre-treatment with 100 nM CMA had no effect on Ca2+ oscillations in ERf (left) and ET (right). (TIF) [file pone.0039499.s004.tif]

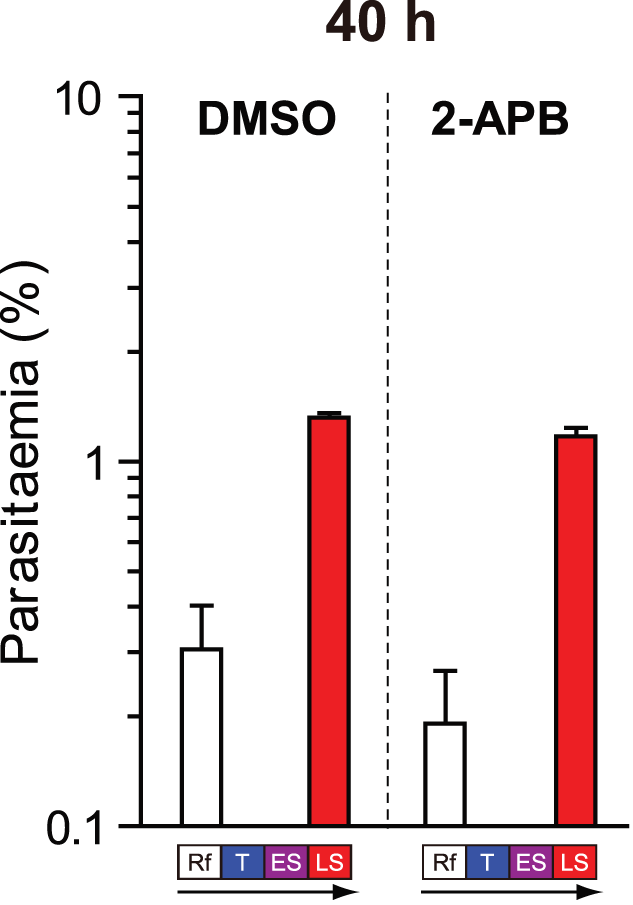

Supplement: Figure S5 — Pre-treatment of erythrocytes with 2-APB did not inhibit intraerythrocytic development of P. falciparum . To evaluate the effects of 2-APB on host cells, erythrocytes were pre-treated with 100 µM 2-APB for 1 h at 37°C, washed with RPMI medium and resuspended at a haematocrit of 5% in complete culture medium. Cultures of late schizonts (LS; 0.5% parasitaemia) were diluted four times with pre-treated erythrocytes, and the culture was continued. Cultures (three wells per experimental group) were terminated at 40 h of the assay, and thin smears of erythrocytes were prepared for parasite counting. Parasitaemia with ring forms did not differ significantly between dimethyl sulfoxide (DMSO)- and 2-APB-pre-treated groups (P = 0.408, two-tailed unpaired t test). Parasitaemia is shown as mean + S.D. of three independent counts of three wells. Stages with parasitaemia of less than 0.1% are not shown. (TIF) [file pone.0039499.s005.tif]

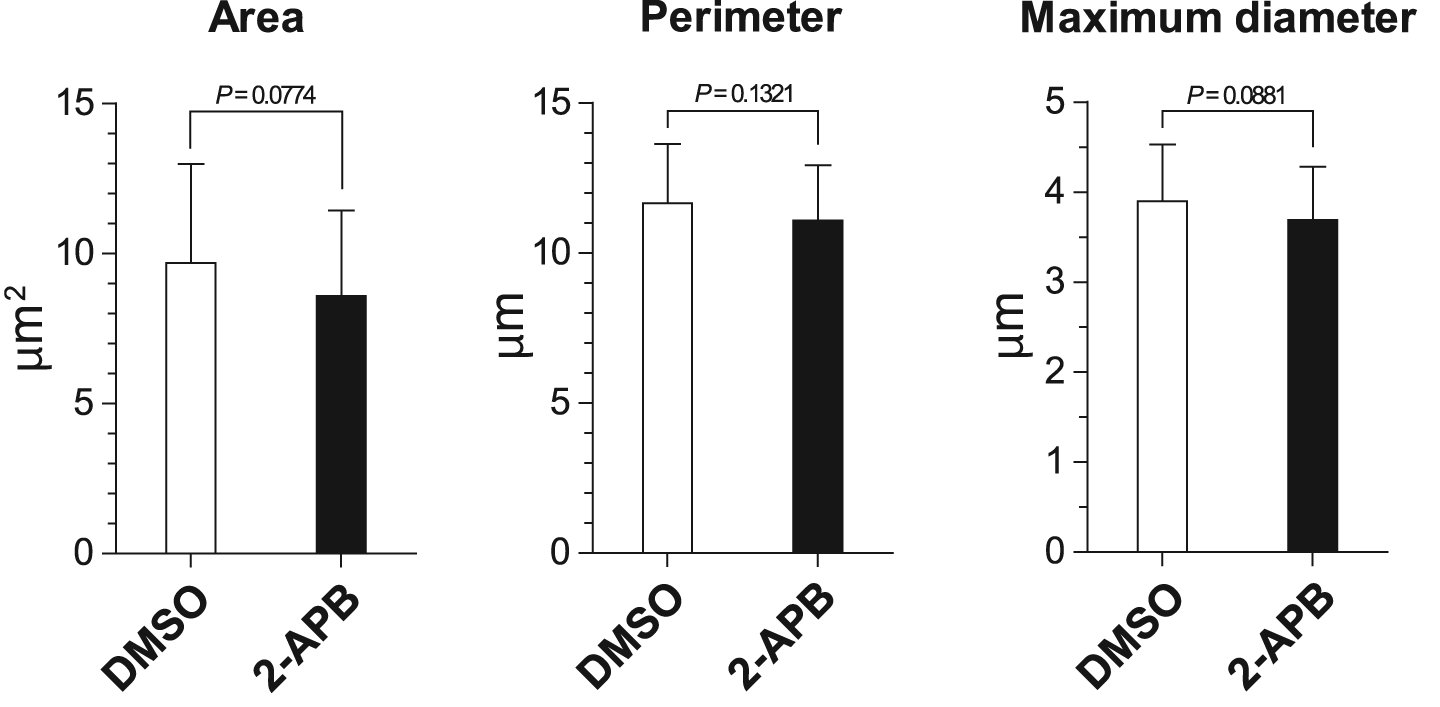

Supplement: Figure S6 — Effect of 2-APB on the area, perimeter and maximum diameter of the chloroquine-resistant strain K1. No statistically significant difference was observed in the area, perimeter and maximum diameter of intraerythrocytic parasites between DMSO- and 100 µ M 2-APB-cultured groups after 24 h of the assay, but the 3 parameters showed a tendency to decrease. Error bars represent mean + S.D. (n = 50). P values are given in each panel (two-tailed unpaired t test). (TIF) [file pone.0039499.s006.tif]

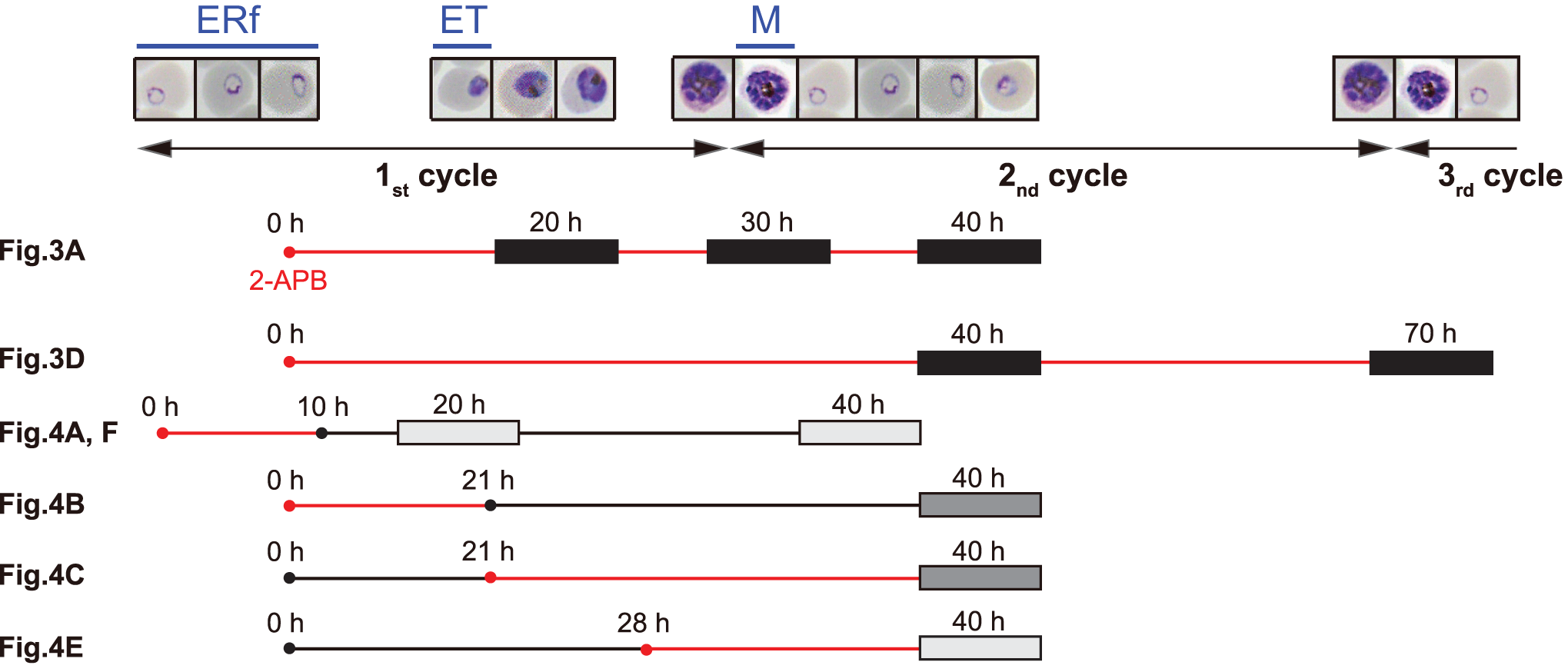

Supplement: Figure S7 — Timetable of the effects of 2-APB on Ca2+ dynamics and parasite development in the blood stage of P. falciparum . Images show Giemsa-stained parasites. Blue bars above the parasite images indicate stages of the parasites in which spontaneous Ca2+ oscillations or small periodic Ca2+ fluctuations were observed. Red lines represent the period of 2-APB treatment. Red circles indicate the initiation of 2-APB treatment. Black, dark and light grey boxes show the extent of the effect of 2-APB during each sampling period: black, severe effect with developmental delay and abnormal morphology; dark grey, weaker effect with developmental delay and abnormal morphology than that when parasites were exposed to 2-APB for 40 h; light grey, slight effect with developmental delay or abnormal morphology compared to that when parasites were exposed to 2-APB for 40 h. (TIF) [file pone.0039499.s007.tif]

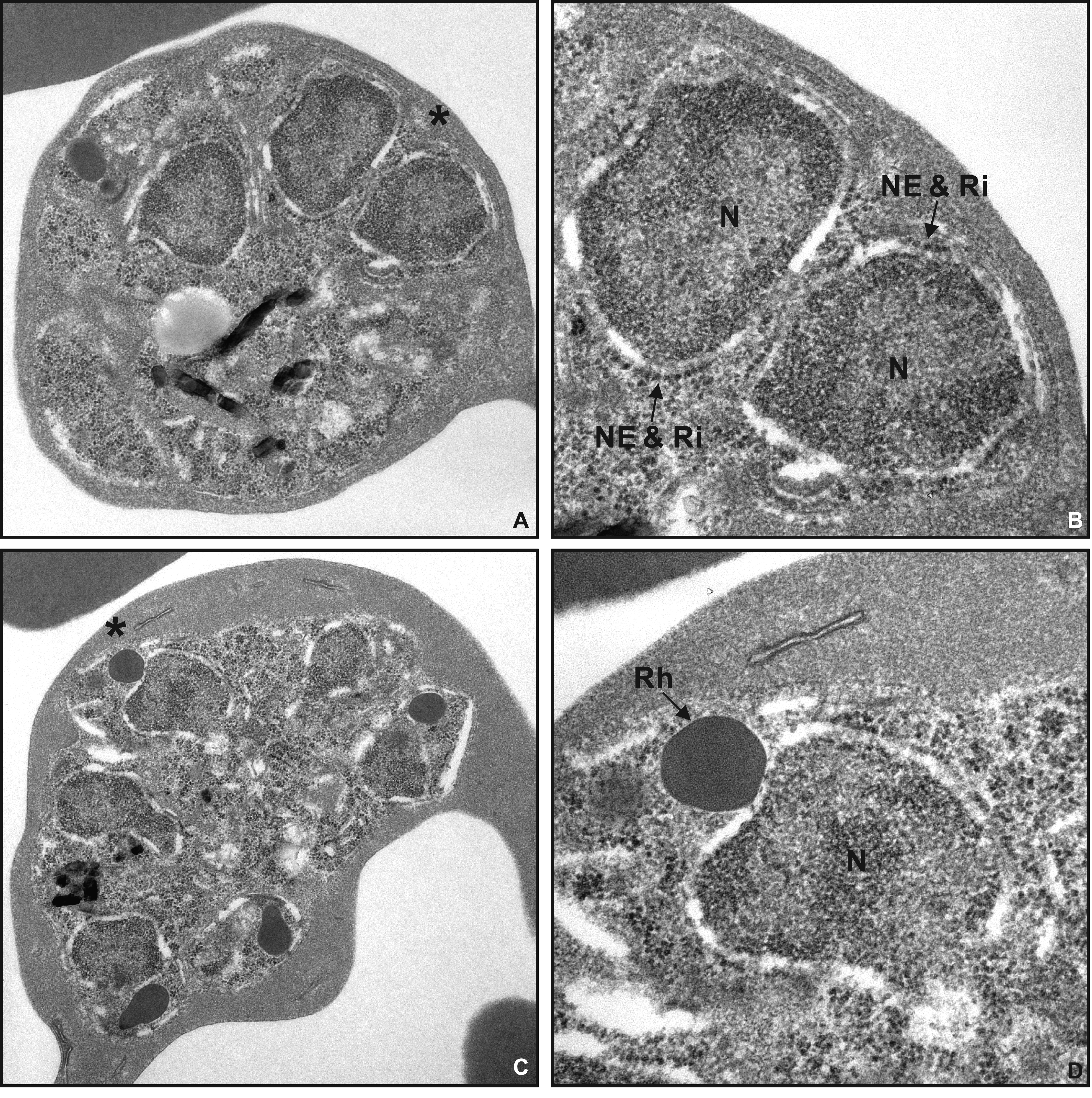

Supplement: Figure S8 — Electron micrographs of parasites in DMSO control culture. (A) At 30 h of the assay (original magnification, ×30,000). (B) Higher magnification image of the part marked with an asterisk in (A) (original magnification, ×80,000). (C) At 30 h of the assay (original magnification, ×20,000). (D) Higher magnification image of the part marked with an asterisk in (C) (original magnification, ×80,000). N, nucleus; NE, nuclear envelope; Ri, ribosome; Rh, rhoptry. (TIF) [file pone.0039499.s008.tif]

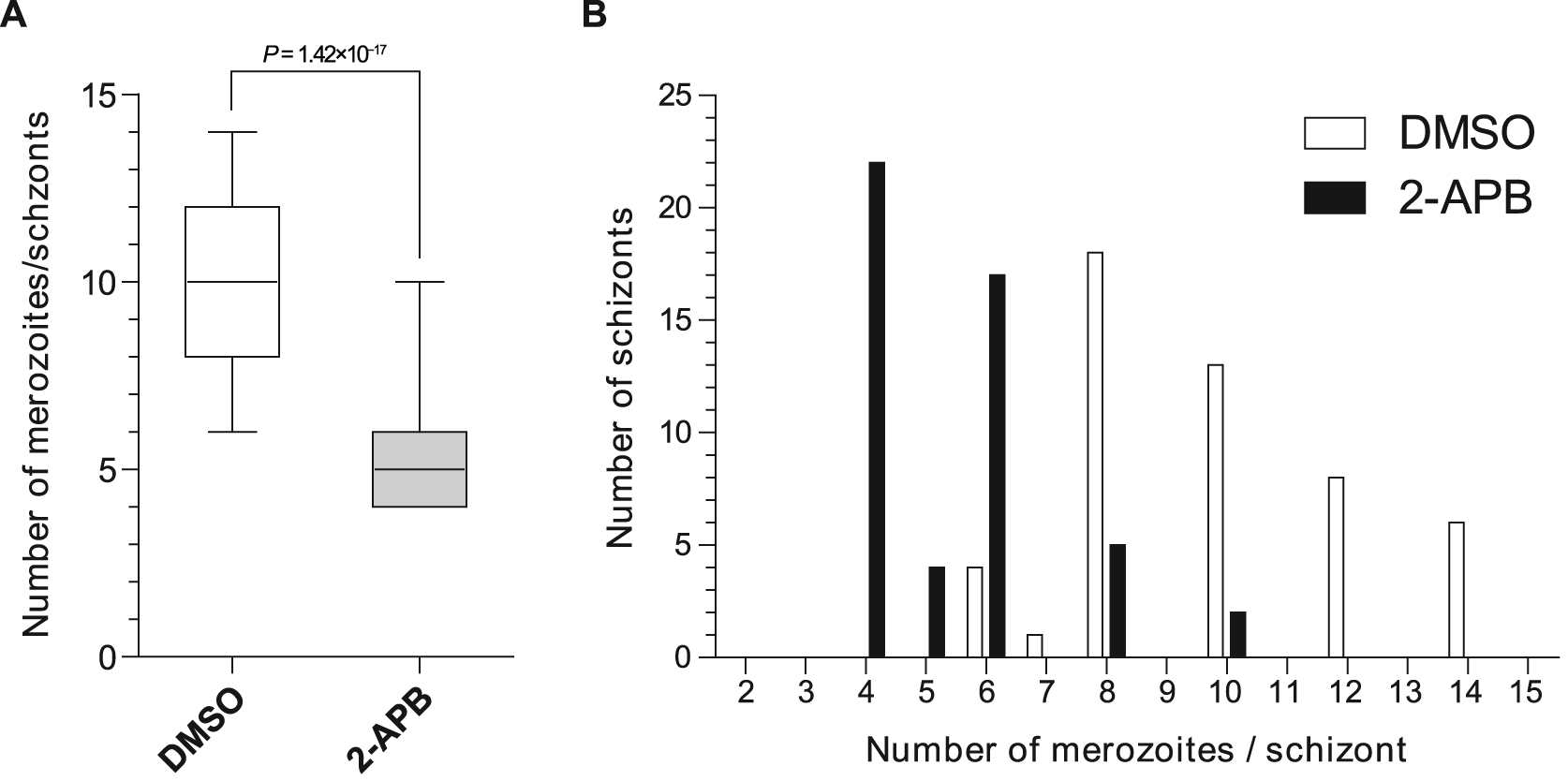

Supplement: Figure S9 — Effects of 2-APB on the number of merozoites in each schizont. (A) Box plot of the number of merozoites (M) formed in each schizont (S) after 40 h of culture with DMSO (white boxes) or 2-APB (light grey boxes). The central rectangle spans the first quartile to the third quartile. The segment inside the rectangle shows the median and whiskers above and below the box show the minimum and maximum. 2-APB significantly decreased the number of M per S (two-tailed unpaired t test with Welch’s correction). (B) Frequency distribution of the raw data shown in (A). (TIF) [file pone.0039499.s009.tif]
